# A Mobile App to Optimize Social Participation for Individuals with Physical Disabilities: Content Validation and Usability Testing

**DOI:** 10.3390/ijerph18041753

**Published:** 2021-02-11

**Authors:** Dahlia Kairy, Mir Abolfazl Mostafavi, Catherine Blanchette-Dallaire, Eva Belanger, Andrea Corbeil, Meena Kandiah, Tian Qiang Wu, Barbara Mazer

**Affiliations:** 1 School of Rehabilitation, Faculty of Medicine, Université de Montréal, Montreal, QC H3N 1X7, Canada; 2 Center for Interdisciplinary Research in Rehabilitation of Greater Montreal, Montreal, QC H3S 1M9, Canada; barbara.mazer@mcgill.ca; 3 Département des Sciences Géomatiques, Faculté de Foresterie, de Géographie et de Géomatique, Centre de Recherche en Donnée et Intelligence Géospatiales, Université Laval, Quebec City, QC G1V 0A6, Canada; mir-abolfazl.mostafavi@scg.ulaval.ca; 4 Center for Interdisciplinary Research in Rehabilitation and Social Integration, Quebec City, QC G1M 2S8, Canada; 5 OnRoule.org, Montreal, QC H2L 1S5, Canada; catherine@onroule.org; 6 School of Physical and Occupational Therapy, Faculty of Medicine and Health Sciences, McGill University, Montreal, QC H3G 1Y5, Canada; eva.belanger@mail.mcgill.ca (E.B.); andrea.corbeil@mail.mcgill.ca (A.C.); meena.kandiah@mail.mcgill.ca (M.K.); tian.q.wu@mail.mcgill.ca (T.Q.W.)

**Keywords:** social participation, mobile application, mHealth, usability, user-centered design, accessibility, physical disability

## Abstract

Background: Social participation is beneficial for individuals’ health. However, people with disabilities that may lead to mobility limitations tend to experience lower levels of social participation. Information and communication technologies such as the *OnRoule* mobile application (app) can help promote social participation. Objectives: To obtain potential users’ perceptions on the usability and content of the *OnRoule* app for providing information on accessibility, as well as its potential to optimize social participation. Materials and Methods: Cross-sectional user-centered design study. Individuals with physical disabilities (*n* = 18) were recruited through community organizations and interviewed using a semi-structured guide. Interviews were recorded, transcribed, and analyzed using thematic analysis. Results: Three main themes were identified: (1) “user-friendliness”; (2) “balance between the amount and relevance of information”; and (3) “potential use of the app”. Discussion and Conclusion: Findings from this study indicated that the app was easy to use, had pertinent information, and enabled a positive experience of finding information. However, several areas of improvement were identified, such as the clarity of specific elements, organization and amount of information, optimization of features, and inclusiveness. Apps such as *OnRoule* could optimize social participation by facilitating the process of finding resources in the community and building a sense of connectedness between users.

## 1. Introduction

Social participation is defined as an individual’s engagement in activities that incorporate interaction with others in society or within the community [1]. Activities promoting social participation include work, volunteering, education, and leisure activities that engage the participants in physical or artistic pursuits [2]. The benefits of social participation are well documented, making it an important goal in rehabilitation. In fact, social participation has been linked to higher levels of self-reported health and described as an indicator of well-being [3,4,5]. Social participation provides emotional support, personal fulfilment, and information about healthy lifestyles while protecting from isolation [3,6].

Individuals with disabilities experience lower levels of social participation [7]. They are less likely to belong to a group, to engage in political activities, or to feel like they are part of their local community [8,9,10]. Several factors can limit their level of social participation, such as functional limitations, limited environmental accessibility, lack of information on resources, and stigma associated with disabilities [11,12,13,14]. Functional limitations can be addressed with remedial approaches or compensatory strategies [15], and environmental access may be facilitated through physical adaptations such as ramps and elevators [16]. One approach to addressing societal attitudes toward individuals with disabilities is through public awareness campaigns [17]. Finally, access to information may be facilitated with the distribution of pamphlets or through online databases [18]. For example, providing information on the social and physical accessibility of resources, the availability of adaptive equipment and the option of obtaining attendant care during an activity could increase an individual’s participation in the community [19]. Web-based information has the advantage of being generally more up-to-date, more diversified and readily available. In addition, while some locations and activities may be accessible to some individuals with disabilities, they may not be for others, as proposed by the concept of human accessibility (HA) [20]. As opposed to universal accessibility, which aims for locations and activities to be accessible to all levels of disabilities, HA suggests that the perception and capacity of use of the accessibility of a location may change from one person to another, depending on the individual’s mindset and capacities, and may change over time, depending on the individual’s health level and type of mobility device used. Therefore, the “accessible” status of a location is related to the individual and changes from one person to another. Hence, the information provided allows people of various capacity levels to find information that is relevant to their own specific needs, which may not be relevant to everyone.

Information and communication technologies (ICTs) are an emergent area of research focused on the development of software that can be used to retrieve and share information on devices such as computers and smartphones [21,22,23]. ICTs attempt to meet healthcare demands in terms of fast information transfer and promotion of patient self-management [24]. Mobile applications (apps) are a common type of ICT and are defined as downloadable programs on the operating system of smart devices to enable the phone or the tablet to fulfil a specific function [24,25]. Apps can be used to empower citizens on important health matters such as social participation [24]. Some characteristics are important to consider in order to make these apps usable and relevant, in particular when designing apps for individuals with disabilities.

An app is considered readily usable when it can be operated intuitively by the user [26]. The following criteria were established to facilitate the usability of an app: the procedures and operations of the app must be efficient, easy to remember, easy to learn and subjectively pleasant for the user, and must entail minimal error [26]. Some features that were found to optimize the users’ experience were rating systems, aesthetic considerations, credible information, various information levels (details), and interactive platforms [25]. For individuals living with disabilities, specific considerations must be made to having a universal design that ensures inclusiveness of all users, including operations that require minimal physical effort, and giving access to customizable information [26]. Hence, these criteria should be acknowledged when designing an app targeting accessibility to community resources for individuals with physical disabilities. In order to ensure that an app addresses the users’ needs, a user-centered approach can be used, such as consulting individuals with particular needs to provide input on the design, implementation, and evaluation of the apps, throughout the app development process [21,25,27,28].

Another pertinent feature that can be integrated in an app is crowdsourcing, which is defined as “the practice of obtaining needed services, ideas, or content by soliciting contributions from a large group of people and especially from the online community rather than from traditional employees [...]” [29]. Crowdsourcing is important in the development and ongoing improvement of mobile software because it allows information to stay up-to-date and relevant for the user [21,25,27], which is particularly relevant for people with physical disabilities who have different or sometimes changing capabilities.

Several local existing apps, such as Jaccede [30], Wheelmap [31], Woussoul [32], AccessNow [33] and Access Earth [34] provide information on the physical accessibility of locations such as restaurants, grocery stores and sporting clubs, and allow users to provide a rating based on their experience. However, these existing resources do not provide the level of detail, such as the availability of adaptive equipment or of support from an attendant, for example, or allow for continued user input and interaction. In addition, they have not been developed using a systematic theory-based process to take into account user needs with respect to accessibility and social participation, as has been suggested.

### The OnRoule App

OnRoule.org is a non-profit organization with a website aimed at filling this gap by giving information on the physical accessibility of locations across Quebec (Canada) and providing additional relevant information regarding the availability of adaptive equipment and other factors which may impact on accessibility [35]. However, compared to a mobile application, a website may not be as convenient and accessible for a mobile user. Therefore, the *OnRoule* app was developed to optimize quick information retrieval and allow spontaneity and facilitate social engagement in meaningful activities for adults with physical disabilities. In collaboration with OnRoule.org, a series of exploratory studies were conducted with potential users and their caregivers (referred to as study 1) in order to determine the appreciation and needs of adults with mobility limitations concerning the *OnRoule* app and its role for social participation. That study identified barriers and facilitators of social participation and obtained user preferences regarding features and content to be included or improved in the app. Based on these findings, feedback was integrated into the *OnRoule* app’s mock-ups of the interface design [36].

The *OnRoule* app aims to provide users with quick access to individualized useful information about the accessibility of a wide array of public locations. Important concepts emerging in the field of accessibility were taken into account in the app’s development. These include developing an app for a wide range of users, regardless of disabilities or mobility limitations, using a user-centered approach, and being proactive in identifying accessibility issues with regards to the app itself and its content [37]. As well, the *OnRoule* app is designed based on the concept of HA—for users to be able to plan and increase their social participation based on their own particular needs and abilities, which are specific and unique to each person, and may also change in time [20]. In order to do so, a wide array of features was made available when the app was first developed based on the website and feedback from potential future users (i.e., study 1), therefore proactively identifying the users’ needs and requirements. For example, geolocalization, once activated, would enable users to find a nearby location. The public locations were classified into different categories such as “shopping”, “food establishments”, “entertainment”, and “courses” in order to facilitate the search process. Locations for people with various types and levels of disability were included, in order for individuals to find information relevant to their capabilities as suggested by the concept of HA. When creating a user profile, the user could select the accessibility parameters of interest. For example, the user can choose to view details only about the entrance, the washroom, and the parking spaces, or choose to see information about the visual and auditory accessibility of the location. Other options of customization offered by the app are related to the colours and contrasts of the app and the size and style of the font.

This app uses a crowdsourcing model that enables users to add information about locations, rate the locations, add pictures, and leave comments that are then made available to other users. Additionally, a social network platform is integrated into the app to nurture a sense of connectivity and to facilitate the retrieval of relevant information by filtering other users that live in the same city or have the same mobility needs, for example.

As part of a user-centered design process, the objective of this study was to obtain the perceptions of individuals with mobility limitations on the content and usability of the app based on user-input, as well as further explore how the app may optimize social participation.

## 2. Material and Methods

A qualitative study design was used to examine potential users’ unique perceptions, experiences, and views on regarding the *OnRoule* app [38]. The research paradigm to conduct this study was consistent with the constructivist perspective, the focus being placed on understanding the subjective reality of each participant [39]. Specifically, an interpretive description methodology was used to obtain information to be used in the on-going development of the app which would be useful for people with disabilities as well as rehabilitation clinicians providing services [40].

### 2.1. Participants

The primary informants were individuals with physical disabilities with mobility limitations who may benefit from such an app. The inclusion criteria were: (1) being 18 years old or older, (2) being able to understand and speak either French or English, (3) being able to read and understand basic terms in French as the app’s interactive mock-ups were at this stage only available in French, (4) being able to consent for themselves, (5) having a self-reported physical disability resulting in a long-term mobility limitation, and (6) owning a tablet or a smartphone and having previously used mobile applications. Exclusion criteria were: (1) having a hearing or visual deficit significantly limiting their ability to participate in the interview and interact with the app, (2) having emotional or psychiatric issues that could limit their participation in the study, and (3) having an intellectual disability that could limit their ability to engage in the study.

Convenience sampling, a method of recruitment founded on the availability and the accessibility of participants [41] was used to sample a group of participants with physical limitations. Participants were recruited from those who took part in study 1 and had given permission to be recontacted as well as from *OnRoule*’s community. Participants were recruited until saturation was obtained as agreed upon by the team when no new information emerged (see data analysis below) [41].

### 2.2. Data Collection and Analysis

Observations and semi-structured interviews were conducted with the participants. Prior to the interviews, socio-demographic and information on participants’ use of smart devices was collected during an initial telephone call. In-person interviews were essential because participants were observed while interacting with the *OnRoule* interactive app mock-ups followed by them responding to interview questions. The face-to-face interviews were conducted by a researcher in French or in English depending on each participant’s preference. An additional researcher was present to observe and take notes using an observation checklist (see Appendix A).

**Figure 1:**
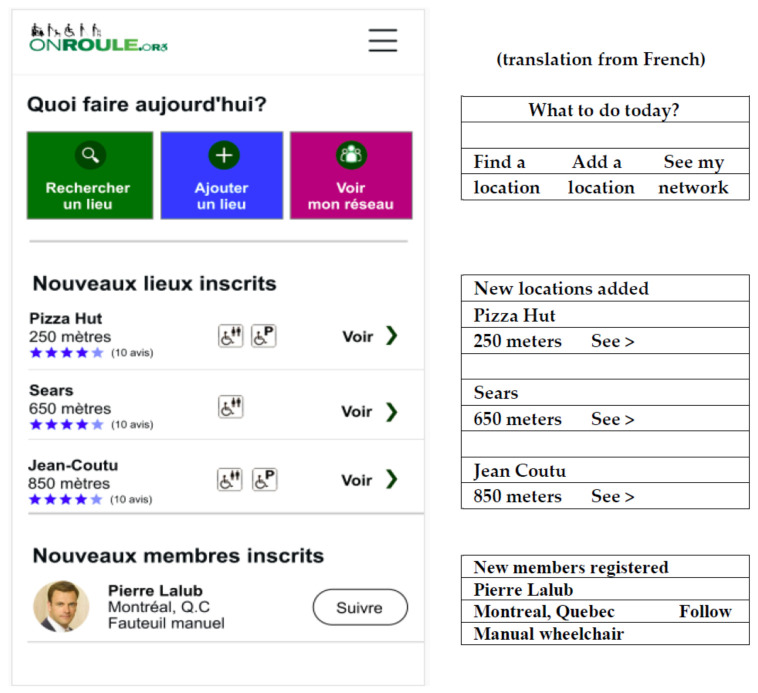
Home page of *OnRoule* app (with translation for publication).

**Figure A1:**
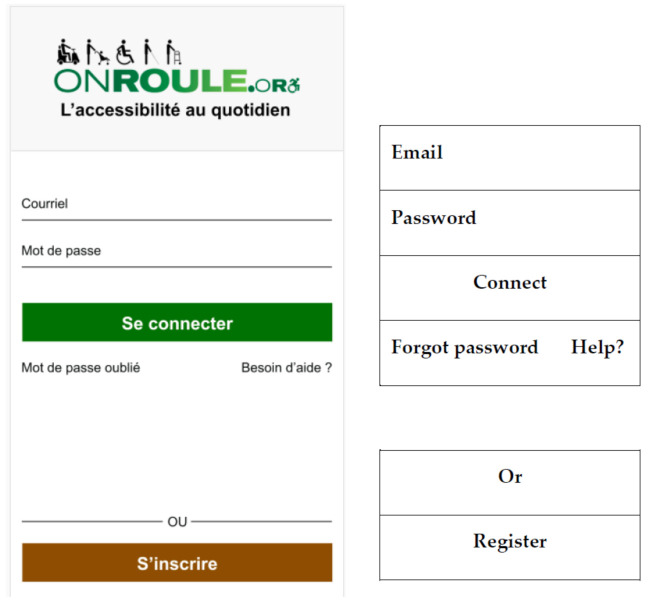
Welcome/Connexion page.

**Figure A2:**
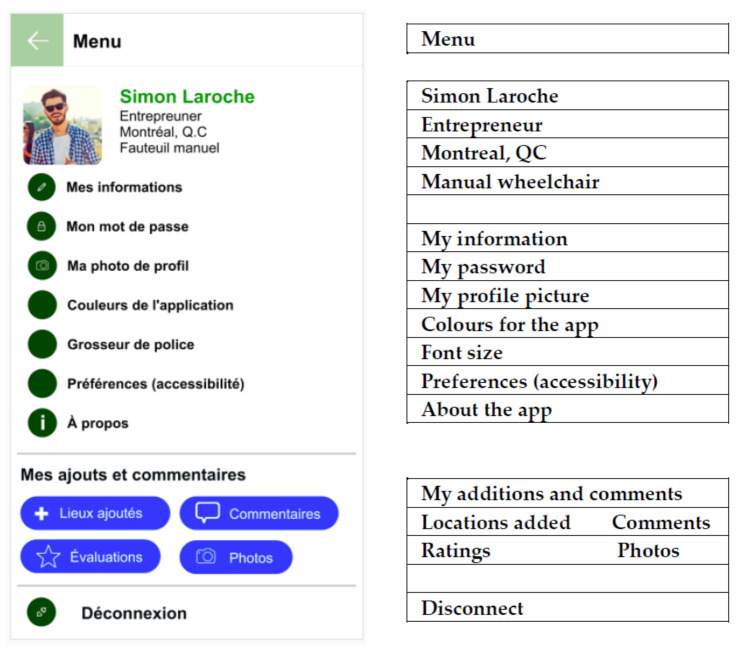
Profile settings page.

**Figure A3:**
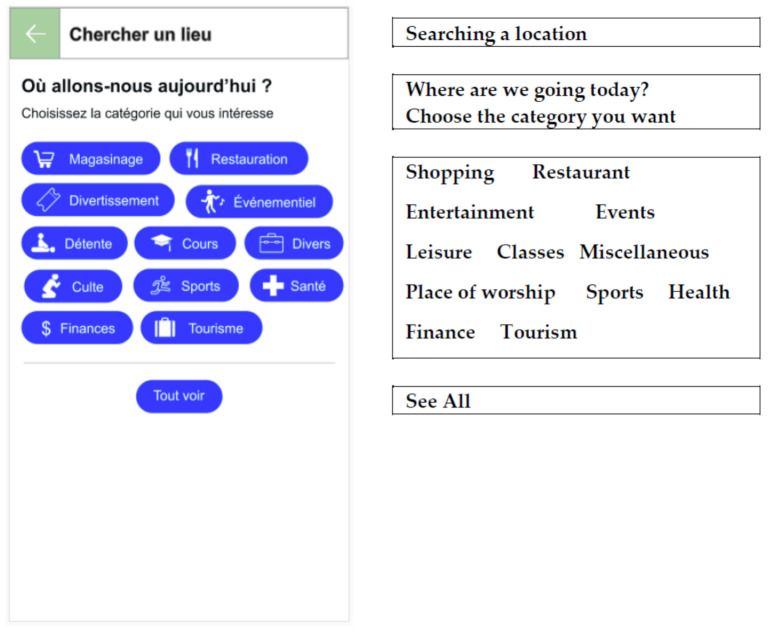
Category page of “Searching a location”.

Using the *OnRoule* app mock-ups (see Appendix B), after accessing the welcome page (Appendix B, Figure A1), five simulated tasks were developed in order to explore the user’s perception regarding the app: creating a user profile (Task 1; Appendix B, Figure A2), searching for a location (Task 2; Appendix B, Figure A3), navigating on the home interface and the menu (Task 3; Figure 1), adding a location (Task 4), follow another user (Task 5). In order to quantify the participants’ ease of performing five interactive tasks, an observation checklist was used (see Appendix A). The observer rated each of the tasks as (1) able to do right away; (2) able to do with some difficulty; and (3) unable to do. During the interview, only the main buttons were activated, which allowed participants to select options and navigate the app. However, users could not enter typed information on the different screens (e.g., type in the name of a location) as the interactive mock-up screens were not linked to any databases, but they could verbally express their intentions. Other features not relevant for the tasks were not made available at this time.

In addition, a semi-structured interview guide with primarily open-ended questions was used to facilitate rapport, to probe interesting ideas that arise, and to follow the participant’s interests or concerns [42]. This guide was modified as the interviews progressed in order to better address the emerging themes (interview guide available upon request). First, the opening questions were asked to better understand the participants’ reality. Participants then performed the five interactive tasks using the interactive app’s mock-ups. Following each task, participants were asked to share their impressions on the ease of performing the task. Participants were then asked to elaborate about their impressions related to the features, the interface appearance, content, and confidentiality parameters of the app. Lastly, questions regarding their view on the app’s potential to optimize social participation were asked. The interview guide and observation checklist were tested by the research team on five individuals prior to the start of the data collection.

Interviews took place at the university or at a location convenient to the participant. The interviews were conducted in a closed room in order to provide privacy to the participants and ensure confidentiality. Each interview lasted between 50 and 150 min.

**Figure 2:**
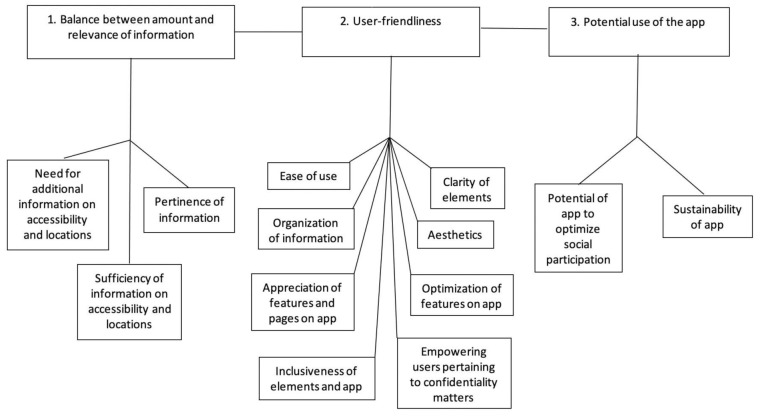
Potential users’ perceptions regarding using an app, such as *OnRoule*, to enhance social participation.

The interviews were audio recorded and transcribed using a naturalized approach, omitting expressions of the oral communication such as “umm” and “ah” [43]. All transcripts were anonymized. Data collection and preliminary analysis of the observations and interviews were conducted concurrently. For each transcript, the ideas were extracted and labelled using individual units of meaning. Members of the research team then regrouped the units into sub-themes and larger overarching themes (see Figure 2). Results were discussed on an ongoing basis among members of the research team. This was particularly relevant given that the members of the research team were from different academic and professional backgrounds and had different levels of experience working with technologies and collaborating with people with disabilities. The observation grids were also reviewed to identify factors which hindered or facilitated use of the app and which corroborated and could help explain the findings.

Prior to the interviews, informed written consent was obtained from all participants.

## 3. Results

Eighteen participants participated in the interviews. Socio-demographic data and information on participants’ use of smart devices can be found in Table 1. The sample included more men than women, with ages ranging from 29 to 73 years. The most common diagnoses of the participants were multiple sclerosis, tetraplegia, and cerebral palsy. Most participants (*n* = 15) used a wheelchair to mobilize at home and/or in the community. Self-reported level of comfort with technology ranged from 2/10 to 10/10, with a mean of 8.06. The rate of participation in activities outside the home averaged 4.9 days per week, ranging from one to seven days per week.

Three main themes emerged in terms of the participants’ perceptions regarding using the *OnRoule* app to enhance social participation: (1) balance between amount and relevance of information; (2) user-friendliness; (3) potential use of the app (see Figure 2). Each of the three main themes are presented in the following section. Verbatim quotes and terms found on the app are included and were translated from French to English by bilingual members of the research team when needed.

### 3.1. Balance between Amount and Relevance of Information

Mixed perceptions were gathered regarding the amount and relevance of information on the app, which points towards the importance of finding a balance between both to satisfy the majority of potential users (Figure 2). Many participants expressed an interest in having additional information on the app while others suggested limiting the app to relevant information.

On one hand, participants expressed the “need for additional information on accessibility and locations”. For instance, participants suggested including additional accessibility information on locations such as the availability of adaptive equipment, the availability of an attendant, the possibility to circulate in the location, the physical measurements of physical spaces, and the floor level of washrooms. When seeing the information available on washrooms on the app, one of the participants asked: “the washroom, is it in the basement? Are there stairs? I hope there are washrooms in the location” (participant 016). Another participant noted that the information provided about locations lacked precision pertaining to the “width of the entrance, the height of door thresholds, and the inclination angle of ramps” (participant 005). Moreover, participants suggested including general information on locations such as the number of washrooms available, the exact cost of parking, the number of parking spaces and information on public transport accessibility.

**Figure A4:**
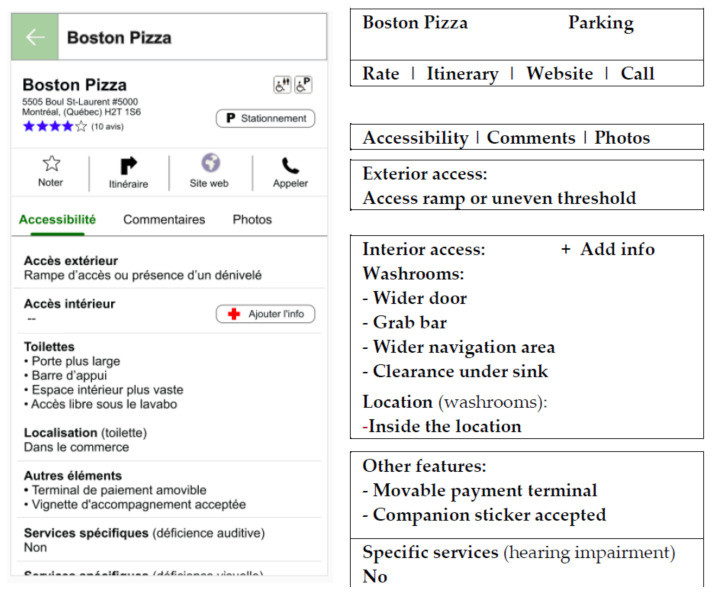
Example of a location page—accessibility data tab enabled (*OnRoule* app).

On the other hand, some participants discussed the “sufficiency of information on accessibility and locations”. They expressed that the information provided was complete and reported a lack of interest in having additional general information on locations as well as additional information on the location’s accessibility (Appendix B, Figure A4). A participant reported: “the content is excellent. You have covered everything” when talking about the content of the location’s page (participant 015). Another participant said: “It is not mandatory to put the exact cost of parking because it changes with time. It could be long to verify all the time” (participant 006).

In addition, some individuals discussed the pertinence of the information on the app. Indeed, participants found some categories of accessibility information on the app pertinent to their needs, notably the information on washrooms and parking. When seeing the information on washrooms, one participant reported: “It could help in the washrooms to know if I can go under the sink or if there is something that will block me from approaching it” (participant 017). However, some participants found another category of accessibility information called “miscellaneous elements” lacking pertinence.

### 3.2. User-Friendliness

Several elements contribute to making an app user-friendly. The eight elements identified through the analysis are described below (Figure 2).

Participants described the mobile application as being user-friendly when it provided them with an ‘ease of use’. Ease of use was experienced when there was ease of performing a task or ease of browsing on the app. In addition, when the app provided sufficient guidance to the user, “ease of use” was optimized. For example, adding infotips, which are small pop-up windows that describe the object being pointed to [44], was a recommendation proposed by a few participants in order to clarify location categories or accessibility options. Moreover, optimizing the graphic design to facilitate navigation was found to be important for the app to be easy to use. For example, “there could be a button to return to the homepage quickly. It would be more helpful” (participant 018). Rapidity of use was also an important factor. More specifically, participants enjoyed when a task was rapid to perform such as the task of adding a location as highlighted by a participant’s statement: “Not too many things to write with the thumbs, it’s just you click, you click. Well done” (participant 011).

‘Clarity of elements’ on the app such as terms, formulations, and icons were important components for the app to be pleasant and convenient to use. Several icons were found to be unclear such as the icon for accessible washrooms or the icon indicating an added location by another user. Some participants suggested adding an icon directory to facilitate understanding: “Maybe at the beginning, when I signed up, it could be interesting to say what the icons mean” (participant 011). Moreover, some formulations lacked clarity. For example, the question “What information do you need?” was perceived as “really ambiguous” (participant 017) and “too vague” (participant 013).

Participants also highlighted the value of organized information on the app. Notably, they identified the need to optimize the classification of information. As an example, finding information through the comments section was seen as a tedious task. Therefore, participants suggested the comments may be filtered based on the type of comment (e.g., accessibility, ambience, quality of service) or based on the mobility aid used by the person who added the comment. They also suggested organizing pictures of locations based on the type of picture (e.g., interior or exterior). Additionally, participants observed that some elements lacked congruency. Within a series of options to choose from, some options were related to accessibility, while others were related to disabilities as described by a participant: “Visual and auditory deficits [options] are not related to the other choices” (participant 017).

Aesthetics of the app was a recurring sub-theme. In fact, the app was found to have an appealing graphic design which was appreciated and consisted of appealing colours, font and layout of information, for example. On the other hand, one participant perceived the app as having an unappealing graphic design, stating that he thought the app was “a bit plain” (participant 009). Participants also had mixed perceptions pertaining to the option of customizing the app’s interface appearance (Appendix B, Figure A2), such as colours and text size. Some participants were interested in these customizable options, some had no personal interest, while others were able to see the pertinence of such features for other users: “I would not use the colour modification function [but] it is useful for other users” (participant 001).

Participants had mixed perceptions in terms of their appreciation of the pages and features on the app. The majority of participants liked the features on the locations’ page such as comments on visited locations, pictures of locations and the itinerary. They also enjoyed the method used to search locations, add locations and follow other users. When searching for other users to follow, users have the possibility to filter by mobility aid or by city. The mobility aid filter function was received more positively as expressed by a participant: “[Filtering by mobility aid] can help because a motorized wheelchair does not pass to the same place as a person with a cane [...] [but] it does not change much where the person lives. There are people from elsewhere who come to Montreal” (participant 017).

Although some features were appreciated, participants shared the need to optimize some of the features on the app. In fact, optimization of searching features was a recurring theme. Most of the participants suggested having a search bar to find locations or to find other users (Appendix B, Figure A3). They also suggested adding the option of searching locations by city: “If I want to prepare in advance and I am looking for a place to swim in [Montreal location], I would like to search by city and not only by geolocalization” (participant 011). Participants also noted the importance of keeping features relevant. They suggested keeping a limited number of features that focus on the main purpose of the app. When proposed the option of adding an instant messaging feature on the app, some questioned the need for a chatroom since other apps can respond to that need. Yet, more than half of the participants were interested in a chatroom and perceived this feature as a possible means to socialize and connect with other users.

Inclusiveness of elements on the app such as graphic design, profile options and accessibility options were thought to be essential for individuals with mobility limitations. In fact, participants suggested adding more accessibility options on the app such as a swipe back feature and a vocal command feature. In terms of graphic design, they felt the text size was small and could be improved to be more inclusive.

The ease of use of the *OnRoule* app was also determined by observing the participants’ performance on the various interactive tasks. For tasks one (create a user account), two (search a location), and four (add a location), the majority of participants were able to do all actions right away, which suggests the app was generally easy to use for these specific tasks. Although the task of searching for a location by categories was easy to perform, participants (*n* = 11) shared the need to add a search bar in order to optimize the searching process. On the other hand, participants had difficulty performing task three (navigate on homepage and menu) and task five (follow another user). When navigating on the homepage and menu, almost half of the participants (*n* = 8) had difficulty finding the menu. In order to access the menu, participants had to click on the “hamburger” button, which is a button placed in a top corner of a page consisting of three horizontal lines. Although frequently used in other apps, some participants (*n* = 5) thought this button lacked clarity, which most likely contributed to their difficulty performing the task. In fact, several participants suggested the addition of the term “menu” beside the button to improve clarity. Also, when attempting to follow another user, more than half of the participants (*n* = 9) did not know they had to click on “my network”. They reported that the term lacked clarity, which made the task difficult to complete.

Finally, participants wanted to feel empowered pertaining to confidentiality matters. They had mixed opinions in terms of openness to share personal information and openness to being followed by other users and following other users. However, they wished the app had more transparency in terms of confidentiality parameters and were interested in having the ability to customize these parameters. In other words, they wanted to know what personal information was being shared with other users of the app and desired some form of control.

### 3.3. Potential Use of the App

One element that stood out as important was envisioning the future development of the app and its potential impacts. Two subthemes emerged, namely ensuring the sustainability of the app, and considering its potential to optimize social participation (Figure 2).

In relation to app sustainability, participants expressed their impressions about strategies to ensure the viability of the app. One recurring idea was the importance of accessing accurate information. Erroneous information would discourage further use of the app. Participant 004 emphasized the value of having other users validate the accessibility of a location through the comment feature: “Nothing better than having a comment from someone who has lived it in a wheelchair”. Moreover, many participants suggested strategies to keep information accurate. Examples were having an external source verifying the validity of the shared information and allowing users to modify information that may have changed over time.

Since the app is based on the principles of crowdsourcing, the contribution of information by users is extremely important. Different motivators to adding information were suggested during the interviews. Most participants agreed that the feeling of benevolence motivated them to add their contribution to the app. Also, making the process of adding data short and easy for users made participants more inclined to do so. Participant 008 clearly stated that “…if you want users to be involved, you have to make it really easy for them”. Participants had mixed perceptions about some strategies meant to motivate them to supply data to the app. Pop-up messages appearing on users’ screens were either liked or disliked by participants. Some participants stated that they would appreciate them if they appeared only sporadically, if they could be deactivated at users’ convenience, or if these pop-ups were messages thanking them for adding information rather than reminders to add information. Other incentive strategies also received various responses: on the one hand, some participants liked the idea of a special user status distinguishing users that were exceptional contributors. On the other hand, some participants did not believe that a special user status would prompt them to add information. Opinions of participants also varied regarding a reward system meant to bestow a monetary compensation like a gift card to those who are active contributors.

The subtheme “potential of the app to optimize social participation” groups the different views of participants regarding how this app could impact their lives. A major asset of the app seems to be its capacity to provide a positive experience of finding information. In fact, participants thought information will be retrieved more quickly and effortlessly when using the app. According to participants, the app could help bridge the gap pertaining to the availability of accurate information about accessibility with respect to their levels of disability. Participant 017 expressed her frustration regarding inaccurate information retrieved from other sources: “I often call restaurants that tell me it is accessible and finally it is not”. Moreover, many participants shared their difficulties when planning and participating in activities outside their home. Therefore, participants shared their hope that the app will facilitate these activities and the associated planning. They opened up about the redundancy of some of their outings: “It’s dull that I do the same things in the same places” (participant 007). In this regard, participants hoped to discover new locations and broaden their opportunities by using the app. Finally, participants were optimistic that the app would create a sense of connectedness between users through the use of the social networking feature, including a chat function.

### 3.4. Observation of Tasks

**Figure 3:**
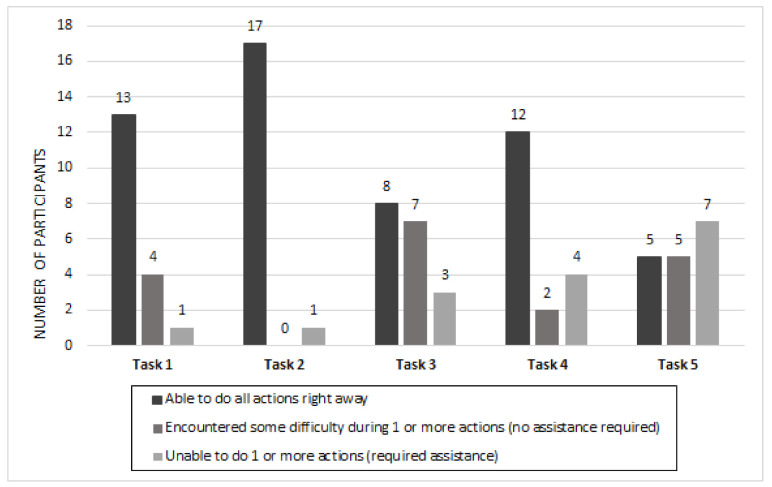
Observational analysis of interactive tasks with app.

Participants were observed while completing the five tasks and their performance was rated using an observation checklist (Appendix A). For task one, create a user profile, the majority of participants (13 out of 18) were able to do all the actions required to create a user profile right away. A few participants (4 out of 18) encountered some difficulty performing the task and one participant was unable to do the task fully. For task two, search a location, almost all participants (17 out of 18) were able to do all the actions in order to find a location right away. For task three, navigate on homepage and menu, less than half of the participants (8 out of 18) were able to navigate the homepage and the main menu effortlessly. Seven participants had some difficulty while three participants were unable to do one or more parts of the task. For task four, add a location, the majority of the participants (12 out of 18) were able to add a location right away. Two participants encountered some difficulty in one or more actions of the task while four were unable to do one or more sections of the task. For task five, follow another user, less than a third of the participants (5 out of 17) were able to follow a user right away. Five participants had some difficulty with one or more sections of the task while seven were unable to do one or more parts of the task (see Figure 3).

## 4. Discussion

In the present study, content and usability of the *OnRoule* app, as well as its relevance to enhance social participation were explored by documenting the perceptions of individuals with physical disabilities and from observations of them performing several tasks with the app.

### 4.1. Validation of Content and Usability Using a User-Centered Design

Several studies have demonstrated the importance of incorporating a user-centered design approach in order to optimize the sustainability of new technologies [25,45,46]. Failure to do so can result in costly redesign of the app and non-usage [47]. The present study, as part of user-centered design process, can inform developers about users’ impressions and needs, allowing them to develop an app tailored to their specific needs. Recommendations about usability and content were described in depth by users based on their first-hand experience of accessible and non-accessible locations. Their rich descriptions provided the research team with innovative design ideas for the continued development of the app and future apps aimed at enhancing social participation.

Comparable studies have been published in recent years. Prémont et al. [48,49] have conducted a scoping review on the usability of the geospatial assistive technologies for the navigation of wheelchair users in urban areas. This study identified a set of usability elements (e.g., information content, interface characteristics, communication modalities, etc.) that help better guide the development of such technologies to manual wheelchair users. Auger et al. [25] examined the usability and content of mobile apps designed to optimize participation at the shopping mall in people with mobility restrictions. Traits of the apps were evaluated using users’ perspectives, similar to the present study, as well an input from an accessibility expert. Similarly, Mayordomo-Martínez et al. [50] examined the usability of ACCEDE Murcia app, which offers information about the accessibility of shops in the city of Murcia, Spain. Their usability audit was performed by an expert. Despite the differences in methodology, similar themes emerged from these studies in terms of content and usability.

Participants in the present study found the information provided by the app to be pertinent. More specifically, they thought the categories of accessibility information were useful (e.g., toilet accessibility, parking accessibility). Similarly, in the study by Auger et al. [25], users found the information on the app relevant (e.g., presence of elevator or stairs). These findings confirm the relevance of including information presented for individuals with mobility limitations.

Additionally, similar to findings from Auger et al. [25], participants in the present study also reported the importance of ensuring the content is not vague, incomplete or lacking precision. Moreover, the importance of precision of information was an impression shared amongst most participants of the present study. For example, participants reported the need for precise measurements of physical spaces. These findings emphasize the importance for the information to be as complete and precise as possible for a population with mobility limitations. This allows individuals to identify locations or activities which are accessible to them even though they may not be accessible to others. This corresponds to the concept of HA [20] on which the *OnRoule* workflow is based, where the range of information provided allows people with various capabilities to find information that is relevant to their own specific needs.

Participants highlighted that there should be a balance between the amount and relevance of information present on the app. Indeed, participants in the present study and in past studies appreciated when a task was easy and quick to complete [25,50]. By including too much information on the app, browsing and completing actions efficiently may be compromised. However, based on HA [20], information should be included that is relevant for various kind and levels of impairments. Therefore, developers must carefully consider this when integrating content in the app.

In this study, ease of use was experienced when a task was easy and rapid to perform, and was important to the app’s usability. Participants preferred having minimal text to enter and being able to select options (Appendix B, Figure A3). Similar findings have previously been reported. For example, in Mayordomo-Martínez et al.’s [50] usability audit, “ease of use”, the ability to navigate the app with ease, was included as an important contributor to usability. Moreover, Auger et al. [25] reported that apps were easy to use when there were a limited number of steps to obtain the desired information. Similarly, in a study examining the mobile experience of smartphone users with motor impairments, about half of the participants found text entry difficult, which highlights the importance of having options to select from on the app’s pages to ensure a positive user experience [51].

Lack of clarity of elements such as icons, and terms (e.g., “miscellaneous elements”, “raised seat”) was a recurring theme in the present study. Similarly, Auger et al. [25] found that vocabulary was sometimes difficult to understand (e.g., “multilevel access”, “visual guidance disposal”). In sum, clarity of elements on the app is essential in order to provide a user-friendly experience and facilitate usage.

Several participants evaluating the *OnRoule* app appreciated the customization options present on the app. Similar to guidelines from the Center for Universal Design [26] with regards to cellular phones, apps should provide alternative methods for operation and be customizable. Several apps have incorporated this principle into their design. In fact, Mayordomo-Martínez et al. [50] found the customizability of the ACCEDE Murcia app could be improved to offer more adaptations based on the preferences of the users (e.g., colour schema, text size). These findings highlight the need to keep the customization options on the final version of the app.

In addition, participants in this study shared the need to optimize the rating system feature consisting of the average of three five-star scales (i.e., accessibility, atmosphere, and service) in order for the rating to be more precise. Likewise, according to Auger et al. [25] participants found a five-star scale lacked precision and clarity. In fact, similar rating systems are present on other apps such as ACCEDE Murcia, where users have the option of rating their accessibility experience at shops from 0 to 5 [50]. However, individuals with mobility limitations seem to agree this type of rating system does not correspond to their need for precise information.

Finally, ensuring the app is inclusive, i.e., allows people with different disabilities to use and participate in the app, is an essential aspect to consider [26]. According to Auger et al. [25], users liked having large characters, as these were easier to read, while participants in our study thought some elements of the app lacked inclusiveness for people of all ages for example, due to the small text size. Inclusiveness must be considered when optimizing the app for it to be usable by all.

### 4.2. Optimizing Social Participation

In the present study, participants shared several ways in which apps such as the *OnRoule* app could potentially optimize social participation. First, participants perceived the app as a means to facilitate activities outside the home by having access to a tool for easier planning. Similarly, in a study investigating how smartphones are being used on a daily basis and what activities they enable in people with motor impairments, participants were found to use apps on smartphones to alleviate physical accessibility challenges such as planning transit [52].

Second, participants thought the app would be useful for them to find information regarding accessibility. They thought the app could enable quicker and effortless access to large amounts of information, and that this information would be more accurate than a traditional paper source of information. In fact, currently, most people expect to access information that is readily available [51]. Similarly, in a 2014 study by Burford and Park [53] that examined the impact of mobile tablet usage on human’s information seeking behavior, the authors found that apps are increasingly used as an access point to all types of digital information and enable access and use of large amounts of digital information without being restricted by location.

Finally, participants appreciated the sense of community provided by the app and perceived the app as a way to create interactions between users. Several users expressed an interest in the addition of a chatroom feature. In a study investigating the impact of internet communication on users’ psychological health, investigators found that engaging in a series of chat sessions with an anonymous partner significantly decreased loneliness and depression, while perceived social support and self-esteem increased [54]. Currently, the *OnRoule* app, allows social networking without a chatroom feature, although the potential benefits associated with adding such a feature are of interest to some participants. Furthermore, perceived accessibility to key resources is a predictor of social participation in older adults [55], suggesting that apps such as *OnRoule* may help counter isolation in this population.

### 4.3. Study Limitations and Future Directions

Several aspects limit the generalizability of the current findings and can inform future studies. In this study, participants shared their perceptions of an interactive version of *OnRoule* app mock-ups and not the final version. Although the mock-ups’ major functions were activated, some features were not functional, and the participants had to verbally state the steps they would take in order to accomplish certain simulated tasks. However, as part of a user-centered process, the interactive tasks enabled participants to share their impressions regarding the ease of use and comment on possible modifications. Future studies could gather perceptions of potential users using the app in a real-life context.

In addition, although the inter-rater reliability of the observation checklist used to analyze the participants’ ease of performing tasks was not evaluated, the methodology used with constant iterative validation during the data analysis stage ensured agreement among team members.

As a step in the user-centered design process, the current study used convenience sampling to recruit individuals with a physical disability. The current sample was not sufficiently heterogeneous to be generalizable to all potential users (e.g., one participant over the age of 65, and no teenagers nor young adults were included, there was minimal variability in the types of mobility aids used, none of the participants used a white cane, crutches, or a guide dog, comfort with technology among participants was high). Indeed, apps such as the *OnRoule* app may be useful for individuals of all ages who have any type of disability that limits accessibility and who might want to improve their social participation. Conducting research using purposive sampling, a method founded on deliberate selection of participants based on their characteristics (e.g., age, gender, languages spoken, type of mobility aid, type of disability, level of comfort with technology, level of social participation, urban versus rural setting) would ensure a more representative sample [56].

Future studies should continue to include potential users with a range of disabilities which can impact on mobility (e.g., visual, hearing, cognitive, intellectual or psychological), as well as various stakeholders such as caregivers, clinicians from different health care settings, representatives of organizations that may be listed in the app or may have members who use the app, as well as accessibility experts [50], to ensure the relevance of such apps.

## 5. Implications

Accessibility apps, such as the *OnRoule* app, which could contribute to increasing social participation and decreasing social isolation, could be part of the tools and strategies used by health care professionals working with people with disabilities. As social participation is a major health determinant and often a long-term goal in rehabilitation, clinicians could use and recommend the app to patients and their caregivers as a means of retrieving information about the available activities within their communities. Being able to personalize the information retrieved by modifying a user profile would allow people with varying levels of disability and individuals whose level of disability will change over time, such as for patients in rehabilitation centers whose abilities may improve, individuals with degenerative conditions or for elderly individuals whose capacities may decline, to continue to optimize their level of social participation over time. By promoting the use of such apps, patients with mobility limitations may feel better equipped with information retrieval and engagement in meaningful activities made easier.

## 6. Conclusions

The aim of the study was to gather the perceptions of individuals with physical limitations regarding an app providing information on accessibility of public places as well as explore the *OnRoule* app’s usability and content, as part of a user-centered design process, in order to ensure the potential users’ needs and preferences are considered and integrated in the final version of the app. In general, the app had a fair usability and content. In fact, the app was overall easy to use, had pertinent and varied levels of information and was viewed as useful for finding information. However, several areas of improvement were identified, such as the clarity of elements, organization of information, amount of information, optimization of features and inclusiveness of the app. Moreover, additional features and content areas were proposed by participants. Finally, future studies should attempt to gather the perceptions from a variety of potential users (e.g., clinicians, caregivers) and explore the use of a functional app in a real-life setting.

## Figures and Tables

**Table 1:** Participant characteristics.

Participant Number	Age	Gender ^a^	Diagnosis	Mobility Aid(s) Used	Self-Report Level of Comfort with Technology (/10)	Rate of Participation in Activities Outside Home (Days/Week)
001	63	F	Amyotrophic lateral sclerosis	Electric wheelchair	7	1–2
002	51	M	Multiple sclerosis	Exoskeleton and cane	9	7
003	36	M	Cerebral palsy	Electric wheelchair	10	7
004	36	F	Partial tetraplegia	Manual wheelchair	8	3–4
005	49	M	Autosomal recessive spastic ataxia of Charlevoix-Saguenay	Manual wheelchair	9	7
006	29	F	Cerebral palsy	Electric wheelchair and 4-wheeled walker	7	3
007	42	F	Paraplegia	Manual wheelchair	7	7
008	46	M	Tetraplegia	Manual and electric wheelchair	8	7
009	73	M	Stroke	None	10	7
010	43	F	Ehlers-Danlos syndrome	Electric wheelchair	7	2–3
011	64	F	Multiple sclerosis	Manual wheelchair	6	7
012	54	M	Unilateral femoral amputation	Manual and electric wheelchair	10	2
013	37	M	Multiple sclerosis	No mobility aid	9	7
014	29	M	Unknown	Manual wheelchair	8	5
015	61	M	Bilateral amputation	Manual wheelchair	2	3
016	60	M	Myasthenia gravis	Manual wheelchair and scooter	10	7
017	42	F	Tetraplegia	Electric wheelchair	10	3
018	55	F	Ataxia	Manual and electric wheelchair	8	2–3

^a^ F = female, M = male.

## Data Availability

The minimal dataset that supports the central findings of this study are available upon request from the corresponding author.

## Appendix A. Observation Checklist *

PARTICIPANT NUMBER: __________________________

INTERVIEWER: _______________________________

OBSERVER: _______________________________

* App items translated from French for publication

**Table A1:** Task 1: Creating a user profile.

	Steps	Able to Do Right Away	Able to Do with Some Difficulty (Errors, Comments)	Unable to Do (Requires Assistance)
**1**	Clicks “Register”			
**2**	Verbally mentions all the information and puts it in the appropriate category			
**3**	Chooses a mobility aid			
**4**	Chooses information on accessibility			
**5**	Clicks “Next” between each page			
**6**	Selects a profile picture or skip step			
**7**	Clicks “Accept”			
**Comments** (e.g., using external resources such as Google, facial expressions, body language):				

**Table A2:** Task 2: Find a place.

	Steps	Able to Find Right Away	Able to Do with Some Difficulty (Errors, Method of Access, Comments)	Unable to Do (Requires Assistance)
**1**	Clicks “Find a place”			
**2**	Clicks “Food”			
**3**	Clicks “Restaurants”			
**4**	Clicks “View” (beside Boston Pizza)			
**5**	Clicks “Comments”			
**6**	Clicks “Photos”			
**7**	Clicks “Parking”			
**Comments**:				

**Table A3:** Task 3: Navigating the homepage and the main menu.

	Steps	Able to Find Right Away	Able to Do with Some Difficulty (Errors, Method of Access, Comments)	Unable to Do (Requires Assistance)
**1**	Returns to “Home Page” (by clicking arrows)			
**2**	Clicks on menu tab (top right)			
**Comments:**				

**Table A4:** Task 4: Adding a location.

	Steps	Able to Do Right Away	Able to Do with Some Difficulty (Errors, Comments)	Unable to Do (Requires Assistance)
**1**	Goes back on “Home Page” (by clicking the arrow)			
**2**	Clicks on “Add a location”			
**3**	Verbally mentions all the information and puts it in the appropriate category			
**4**	Clicks “Next” after each step			
**5**	Clicks on a star for the 3 categories (clicks on appropriate icon)			
**6**	Adds a picture (clicks on appropriate icon)			
**7**	Clicks “End”			
**Comments:**				

**Table A5:** Task 5: Following another user.

	Steps	Able to Do Right Away	Able to Do with Some Difficulty (Errors, Comments)	Unable to Do (Requires Assistance)
**1**	Clicks “Return to Home Page”			
**2**	Clicks “My network” (can receive help to click on the icon)			
**3**	Clicks “See more”			
**4**	Clicks “Follow” beside “Philippine Lalou with guide dog”			
**Comments:**				

## Appendix B. Examples of Pages of *OnRoule* App *

* (translation from French to English provided for publication)
